# Case report: Thyroid carcinoma invading trachea: Multidisciplinary resection and reconstruction assisted by extracorporeal membrane oxygenation

**DOI:** 10.3389/fonc.2022.990600

**Published:** 2023-01-12

**Authors:** Bo He, Shixin Zhang, Lin Ren, Yi Zhou, Qiao Chen, Jinghua Tang, Yi Zhang, Meng Tang, Yang Qiu, Haidong Wang

**Affiliations:** ^1^ Department of Thoracic Surgery, Southwest Hospital, Army Medical University (Third Military Medical University), Chongqing, China; ^2^ Department of Breast and Thyroid Surgery, Southwest Hospital, Army Medical University (Third Military Medical University), Chongqing, China

**Keywords:** ECMO, thyroid tumor resection, trachea reconstruction, case report, safe and effective

## Abstract

**Background:**

When thyroid cancer invades the trachea, tumor resection and trachea reconstruction are required. Although the traditional way of anesthesia and tracheal intubation can maintain the necessary ventilation function during the operation, tracheal intubation affects the surgical field of vision and is not conducive to the protection of the recurrent laryngeal nerve beside the trachea during the operation.

**Case presentation:**

Extracorporeal membrane oxygenation (ECMO) is used to replace traditional tracheal intubation in the process of resection and end-to-end anastomosis of tracheal tumors, and complete tracheal tumor resection and trachea reconstruction are achieved.

**Conclusion:**

Using ECMO for thyroid carcinoma resection, invaded trachea resection, and trachea reconstruction is safe and effective, which reduces the obstruction of endotracheal intubation on the operative field, guarantees the rapid and efficient end-to-end anastomosis in the upper trachea, and clearly avoids laryngeal recurrent nerve injury in the process of anastomosis.

## Background

In 2020, the number of new cases of thyroid cancer in the world was about 580,000, and the incidence of thyroid cancer ranked 11th among all cancers (1). Thyroid tumors are the most common head and neck tumors involving the cervical trachea. For patients with a thyroid malignant tumor that has invaded the trachea, the operation requires that the thyroid and trachea invaded by the tumor are resected according to the scope of surgery, and tracheal respiration and neck dissection should be performed according to the situation (2, 3).

When thyroid cancer invades the trachea and recurrent laryngeal nerve, patients are at risk of respiratory distress, asphyxia, and even death. Sleeve resection of tracheal tumors and airway reconstruction are effective methods to treat tracheal tumors and save patients’ lives. The simultaneous surgery of tracheal resection and tracheal reconstruction is also one of the most complicated and difficult operations, which is required a high level of surgical techniques and perioperative management. The difficulty of the operation is reflected in complete tumor resection, tracheal resection and reconstruction, and retention of functions of the recurrent laryngeal nerve and parathyroid gland at the same time. After multidisciplinary consultation, it was finally determined to complete the operation with extracorporeal membrane oxygenation (ECMO) assistance. This may be the first case of resection and reconstruction of the trachea invaded by thyroid cancer assisted by ECMO in China.

Although the traditional way of anesthesia and tracheal intubation can maintain the necessary ventilation function during the operation, tracheal intubation affects the surgical field of vision and is not conducive to the protection of the recurrent laryngeal nerve beside the trachea during the operation.

Therefore, in order to achieve the best surgical results and further expose the surgical field, we performed resection of thyroid cancer with invaded trachea and tracheal end-to-end anastomosis with the assistance of ECMO without mechanical ventilation and tracheal intubation in the surgical field.

Thyroid surgery was performed with radical thyroidectomy. During the operation, the recurrent laryngeal nerve monitoring technique was used to preserve the function of the invaded recurrent laryngeal nerve. The function of the parathyroid gland was preserved by using hyperfine dorsal membrane anatomy, negative imaging of the parathyroid gland, and parathyroid transplantation so as to ensure the postoperative quality of life.

This study was reported in agreement with the principles of the CARE guidelines (4).

## Case presentation

**Figure 1:**
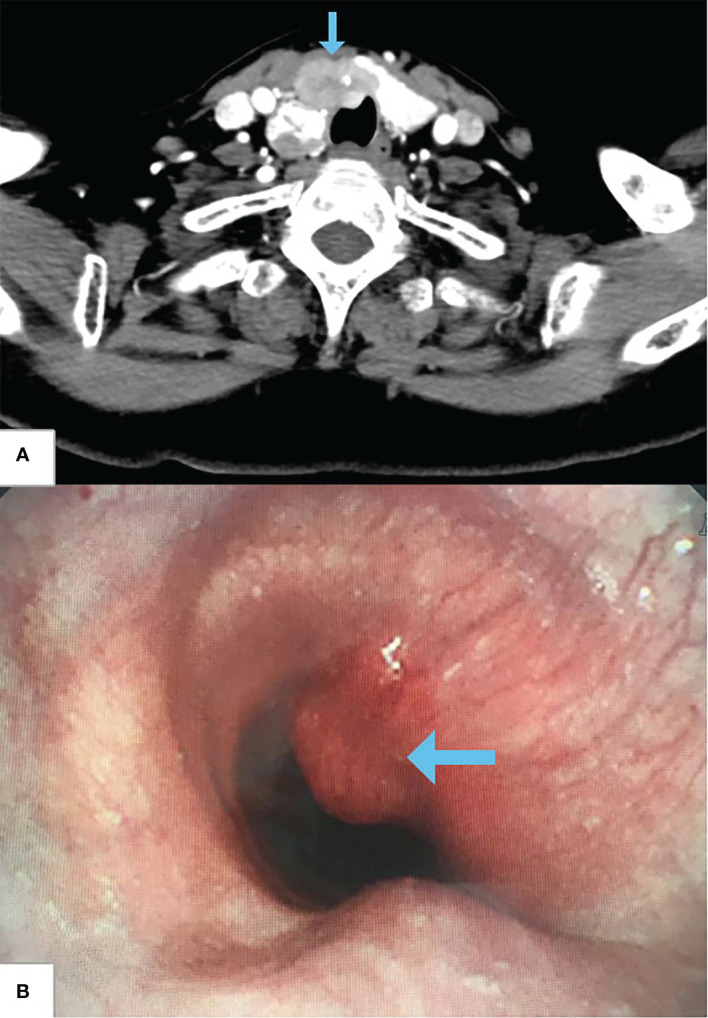
Preoperative cervical CT and fiberoptic bronchoscopy. **(A)** CT shows thyroid carcinoma invading trachea (arrow). **(B)** Fiberoptic bronchoscopy shows a protuberant tumor on the right anterior wall of the trachea, and the tracheal lumen is obviously narrow (arrow).

A 65-year-old woman was referred to our hospital after an examination revealed a right goiter due to an irritating dry cough. Computed tomography (CT) showed a mass in the right lobe and isthmus of the thyroid gland, and thyroid cancer was considered, which invaded the adjacent trachea and was accompanied by lymph node metastasis in cervical region 6 (**Figure 1A**). Fiberoptic bronchoscopy showed a protuberant tumor on the right anterior wall of the trachea, which was about 3 cm away from the glottis; the tracheal lumen was obviously narrow; no obvious abnormality was found in the remaining bilateral bronchus (**Figure 1B**). The cytological results of bronchial brushing showed abnormal cells of which the origin was not excluded from the thyroid.

Before the operation, the multidisciplinary consultation of thoracic surgery, thyroid and breast surgery, anesthesiology, cardiovascular medicine, and respiratory medicine was conducted, and relevant discussions and decisions were completed: 1) thyroid cancer invaded the trachea, and partial tracheal resection and tracheal reconstruction were required during the operation. 2) Preoperative assessment found the patient with mild chronic obstructive pulmonary disease, mild asthma, and airway resistance on the high side, which might lead to endotracheal mass dropping, bronchial foreign bodies blocked, and tumor cell implantation metastasis during induction of anesthesia. In order to ensure surgical safety, ECMO support was strongly recommended by respiratory and anesthesia specialists, which had indications. 3) The neck space was narrow, and the tracheal intubation seriously affected the operation field and hindered anastomosis. With the assistance of ECMO, tracheal intubation could be temporarily removed during intraoperative tracheal resection and reconstruction. 4) For neoplastic lesions with severe airway obstruction, prioritizing the establishment of ECMO during the induction of anesthesia is extremely important to ensure patient safety. In this case, preoperative CT showed that the diameter of the narrowest tracheal area was greater than 1 cm. After preoperative evaluation by the Department of Anesthesiology, it was considered safe and feasible to perform 8-mm outer diameter tracheal intubation. 5) After discussion with the Department of Thyroid and Breast Surgery, it was decided that the operation would be carried out in two stages: the first stage was carried out by the Department of Thyroid and Breast Surgery. After the resection of the thyroid tumor, the scope of tracheal invasion was further explored to accurately determine the way of tracheal anastomosis, minimize anastomotic tension, and also to ensure that the recurrent laryngeal nerve was not damaged during surgery. At this stage, ECMO was not required, and only conventional tracheal intubation was performed. The second stage was performed by the Thoracic Surgery Department. With the surgical field fully exposed under the assistance of ECMO, the Thoracic Surgery Department completed the tracheal tumor resection and end-to-end anastomosis as soon as possible.

After the patient’s informed consent was obtained, the following was performed: total thyroidectomy + bilateral central region lymph node dissection + bilateral recurrent laryngeal nerve exploration + right region 3 and 4 lymph node dissection + left upper parathyroid and left sternocleidomastoid muscle transplantation + ECMO-assisted tracheal tumor resection + tracheal end-to-end anastomosis. The patient was placed in a supine position for general anesthesia. The venous (V-V) ECMO cannulas of the left femoral vein to the right internal jugular vein were quickly established after heparinization.

The detailed operation process is as follows.

The patient was placed in a supine position, and after routine disinfection and towel spreading, 8-mm endotracheal intubation and ventilator ventilation were performed under the guidance of a fiberoptic bronchoscope, and the process was smooth. A 7-cm horizontal low collar incision on the neck was made along the dermatoglyph and 2 cm above the clavicle. Further separation and exploration revealed that the tumor was located in the right thyroid lobe, invading the anterior cervical muscle, the right recurrent laryngeal nerve, and the trachea. The length of the invaded trachea was about 2 cm, and the front wall and both side walls of the second and third cartilage rings were invaded occupying about 2/3 of the trachea, which made it difficult to peel off.

**Figure 2:**
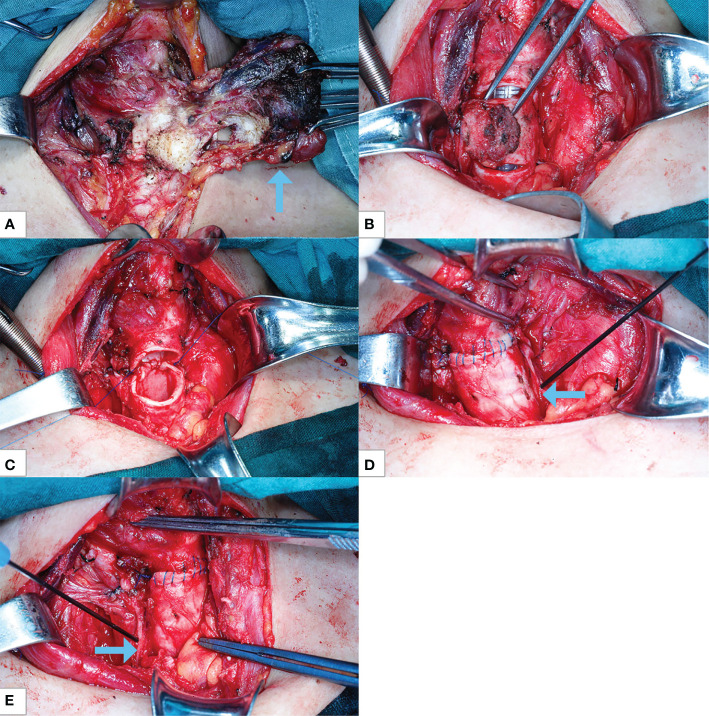
Intraoperative photographs. **(A)** Thyroid cancer invades the trachea (arrow). **(B)** The second and third tracheal rings and tumors are annularly resected with tracheal intubation ventilation condition. **(C)** The trachea end-to-end sleeve anastomosis is ongoing with ECMO ventilation. **(D)** The anastomosis is completed, with left recurrent laryngeal nerve with nerve detection and protection (arrow). **(E)** Right recurrent laryngeal nerve with nerve detection and protection (arrow). ECMO, extracorporeal membrane oxygenation.

The Department of Thyroid and Breast Surgery operated at first. Nano-carbon suspension measuring 0.1 ml was injected into thyroid tissue for parathyroid negative development. The blood vessels of the superior pole of the thyroid were ligated. From top to bottom, the thyroid tissue was separated from the back of the thyroid gland to the surface of the trachea, and the blood vessels of the inferior pole of the thyroid were ligated. The right recurrent laryngeal nerve was seen surrounded by a tumor, and the recurrent laryngeal nerve was separated to be protected by blunt dissection. The upper and lower parathyroid glands were retained, the thyroid tissue was cut off at the isthmus of the thyroid, and the right and left thyroid glands were successfully removed, avoiding the invaded trachea (**Figure 2A**). Then, bilateral central lymph node dissection was performed. During the operation, the monitoring instrument of the recurrent laryngeal nerve showed that the structure of the bilateral recurrent laryngeal nerve was intact, the electrophysiological activity of the left recurrent laryngeal nerve was intact, and that of the right recurrent laryngeal nerve was slightly weakened, which was considered to be caused by tumor invasion of nerve and edema after dissection.

The thoracic surgery team and the ECMO team performed partial tracheal resection and end-to-end sleeve anastomosis assisted by ECMO according to the plan discussed before surgery. Two thoracic surgeons subsequently performed intubation, and one extracorporeal perfusionist prefilled the ECMO tubes and heparinized the whole body. With the assistance of ultrasound, an intravenous cannula (CB96670-021, Medtronic, Shanghai, China) was placed at the opening between the inferior vena cava and the right atrium as an external drainage tube to draw venous blood out of the body, and an arterial cannula (CB96570-019, Medtronic) was placed at the opening between the right internal jugular vein and the right superior atrial vena cava as a return tube to bring external oxygenated blood into the body.

The MAQUET cardiopulmonary bypass package BE-PLS 2050 was used to prefill, exhaust, and connect ECMO tubes and cannulas. ECMO diversion was started, the endotracheal intubation retreated to the vicinity of the tracheal glottis, the ventilator was stopped, and oxygen was supplied by V-V ECMO diversion. Intraoperative ECMO parameters were as follows: centrifugal pump was maintained at 3,500 revolutions per minute, ECMO flow rate was maintained at 3.2 L/min, the fraction of inspiration O_2_ was maintained at 45%, and airflow was maintained at 4.5 L/min. Oxygen saturation during the operation was between 98% and 100%.

The endotracheal tube was retracted near the trachea glottis, and the ventilator was stopped. The second and third tracheal rings and tumors were annularly resected 0.5 cm away from the upper and lower ends of the tracheal tumor (**Figure 2B**). The tracheal tumor specimens were taken out and sent for pathological examination, indicating chronic inflammation of the tracheal stump. The trachea end-to-end sleeve anastomosis of the trachea was completed without tension (**Figures 2C–E**). The monitoring technology of the recurrent laryngeal nerve was used to protect the recurrent laryngeal nerve in the whole process of tracheal resection and anastomosis. The endotracheal intubation was re-sent below the tracheal anastomosis, and the ventilator was resumed for assisted breathing. No air leakage was observed in the anastomosis. The morphology of tracheal end-to-end anastomosis was verified by fiberoptic bronchoscopy.

ECMO was discontinued, ECMO cannulas were removed, activated clotting time (ACT) was examined for 178 s, and 25 mg of protamine was used to neutralize heparin. The circulation time of ECMO was 50 min, during which the patient’s blood pressure was stable and the oxygen saturation was 100%. The operation was smooth and successful, the intraoperative blood loss was about 400 ml, and no blood transfusion was given.

**Figure 3:**
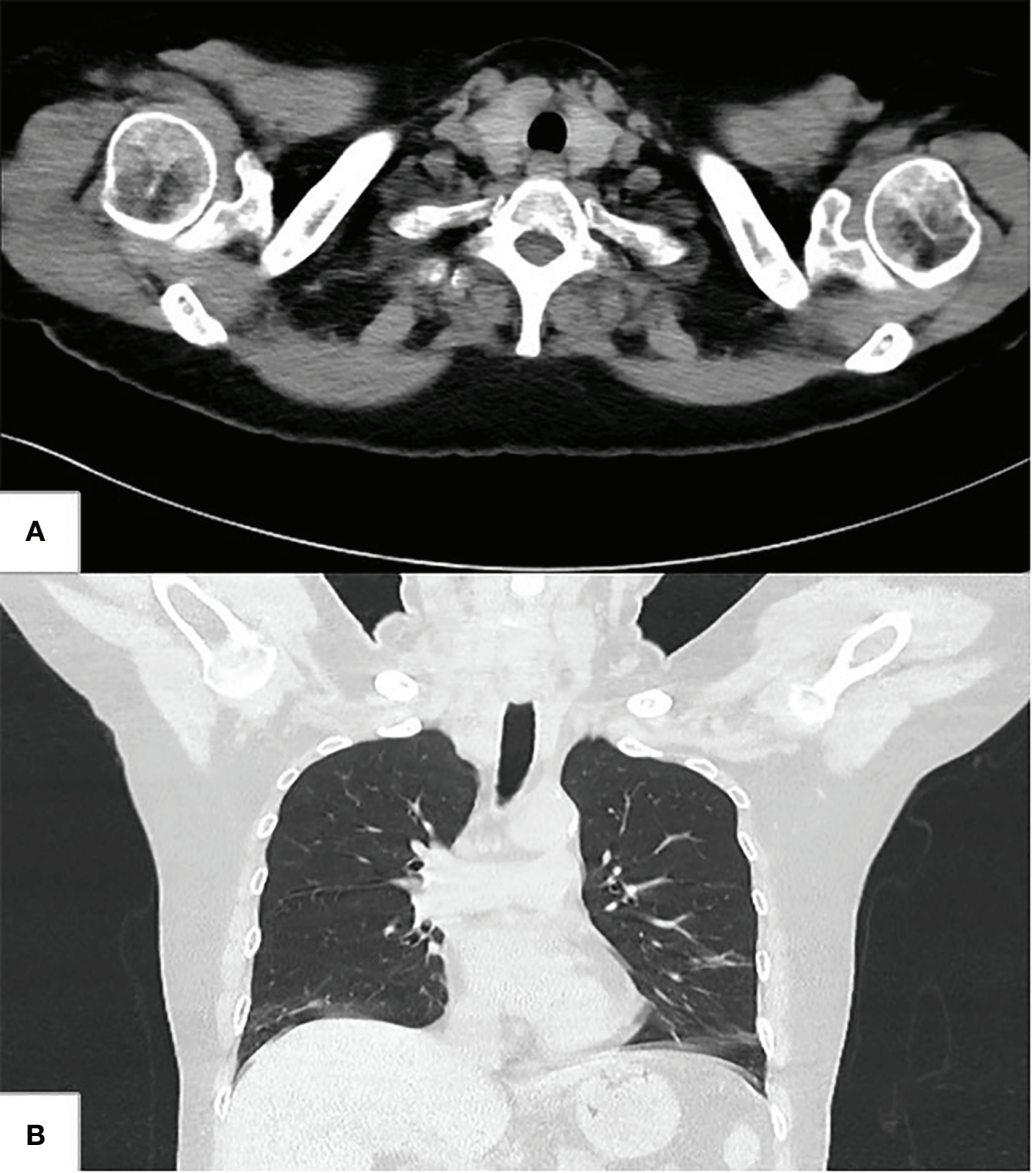
Postoperative neck–chest CT. **(A)** Transverse image shows the right anterior wall of the cervical trachea returned to normal. **(B)** coronal image shows the right anterior wall of the cervical trachea returned to normal.

After the operation, the patient returned to the intensive care unit of thoracic surgery and recovered smoothly. She was discharged from the hospital after 11 days of operation and was required to wear a neck brace and maintain the head-down position for 2 weeks. The symptom of hoarseness was improved. Postoperative pathological results showed that there were three nodules in the right thyroid, two of which were papillary carcinoma involving striated muscle tissue and the whole trachea, and no cancer tissue was involved in the upper and lower stump of the trachea. Metastasis was observed in lymph nodes in the left central region, right central region, and right cervical lateral region. Postoperative neck–chest CT imaging of the respiratory tract showed that the right anterior wall of the cervical trachea returned to normal (**Figures 3A, B**).

## Discussion

Among thyroid cancer, 95% of thyroid cancers were differentiated thyroid cancer (DTC), mainly including papillary thyroid carcinoma (PTC), follicular thyroid carcinoma (FTC), and Hürthle cell carcinoma (HCC). Its clinical treatment involves ultrasound medicine, pathology, surgery, nuclear medicine, endocrinology, oncology, radiotherapy, interventional medicine and laboratory medicine, and many other disciplines. Surgery is the preferred treatment for most DTC patients, which plays a crucial role in the prognosis of the disease. Radical tumor resection is one of the important factors related to the prognosis of patients with DTC. T4 patients with a tumor that has invaded the surrounding structural organs generally are advised total thyroid gland resection; at the same time, the parts of the surrounding affected structures are needed to be removed, like the partial larynx and even the whole larynx, partial trachea, hypopharynx, partial esophagus, and so on. The treatment of DTC is mainly surgical treatment, supplemented by postoperative endocrine therapy, radionuclide therapy, radiotherapy, and targeted therapy in some cases (5).

Different types of thyroid tumors have different degrees and ways of tracheal involvement. The influence of thyroid benign tumors on the cervical trachea is mainly the compression of the trachea, and long compression time may lead to tracheomalacia. Thyroid malignant tumor not only directly compresses the trachea but also invades the trachea. McCaffrey et al. summarized the clinical data of 262 patients with papillary thyroid carcinoma invading the upper respiratory tract from 1940 to 1990 and found that upper respiratory tract involvement was an important factor affecting the survival rate of patients. At the same time, if the upper respiratory tract lumen was invaded, the mortality rate would be significantly increased (6).

Some foreign scholars believe that no matter how much scope of invasion, they are inclined to adopt tracheal circular resection and end-to-end anastomosis. Compared with window resection, this operation completely keeps the prototype of the airway, avoids the occurrence of airway stenosis, and maximizes the negative rate of incision margin. With the improvement of anastomosis technology, the risk of anastomotic leakage was not increased (21% *vs.* 25%), and patients could obtain a greater survival benefit (7).

During the operation, the diseased trachea was not only an important channel to ensure ventilation and maintain oxygenation during anesthesia operation but also the surgical site of surgeons, so the anesthesia management of partial tracheal resection and tracheal reconstruction was very complicated and tricky. There are no anesthetic guidelines and expert consensuses related to a tracheal tumor in China to determine airway opening strategies based on the size, location, characteristics, and degree of ventilation difficulty of tracheal masses (8, 9).

### Method 1

Patients with tumors located in the trachea are generally fit, have a satisfactory cardiopulmonary function, can lie down, have no dyspnea, and have mild tracheal stenosis. Their tumors have wide bases and non-annular growth and do not easily bleed when touched, in which situations rapid induction of endotracheal intubation is feasible. The tracheal tube with a smaller diameter than the narrowest place is selected, and the surface is fully lubricated. Guided by a fiberoptic bronchoscope, the tracheal intubation is completed through the tumor, and the tracheal balloon is placed below the tumor.

### Method 2

Patients have obvious dyspnea and severe tracheal stenosis, their lumen of the narrow section is irregular tubular, and the tumor surface easily bleeds when touched, which is a great risk. Such patients are advocated to keep spontaneous breathing, tracheotomy is conducted with local anesthesia below the tumor, and the endotracheal tube is inserted. Then, the tracheal tube is changed to be inserted through the mouth after resection of the tumor segment and end-to-end anastomosis, and the tracheal tube balloon passes over the anastomotic place.

### Method 3

If the inner diameter of the trachea is severely reduced, the trachea is distorted, or the tumor is free, and the forced insertion of the tracheal intubation will easily cause tumor shedding or bleeding and will cause ventilation dysfunction or suffocation. In this situation, right femoral arteriovenous intubation can be selected under local anesthesia to establish cardiopulmonary bypass and then induce anesthesia. The endotracheal tube is then placed at the proximal end of tracheal stenosis under the guidance of a fiberoptic bronchoscope. During the operation, the trachea is cut open in the lower segment of the stenosis, and tracheal intubation is performed to maintain ventilation. The cardiopulmonary bypass is removed early to reduce related complications such as coagulation dysfunction and intraoperative bleeding.

In 1965, Nevile et al. first reported the application of cardiopulmonary bypass in thoracic surgery (10). However, cardiopulmonary bypass may increase postoperative bleeding and potential complications such as liver and kidney function damage. Moreover, it is technically difficult and time-consuming to establish cardiopulmonary bypass, and at the same time, it can cause great trauma and late infection to patients, so it is not an ideal support method.

### Method 4

ECMO has been recently reported for carinal reconstruction surgery (11). In 2017, our team successfully completed ECMO-assisted tracheal tumor resection and carina reconstruction (Thompson’s operation). We used V-V ECMO to maintain a satisfactory oxygen supply without tracheal intubation, which proved that ECMO-assisted tracheal tumor resection and carina reconstruction is a safer and more effective technique as compared with traditional methods (12). ECMO support has also been used in neonatal trachea reconstruction with satisfactory results (13).

V-V ECMO can support partial or total lung function and can be used in patients with respiratory failure (14). ECMO can completely or partially replace cardiopulmonary function, and its auxiliary application provides the possibility and necessary guarantee for bronchoscopy and surgical treatment, which is a reasonable mode in airway reconstruction surgery.

The ECMO treatment used in this patient has the following advantages: 1) generally, systemic anticoagulation is required during ECMO diversion, which may increase the risk of bleeding during surgery. In this case, ECMO pipelines were established after thyroid tumor resection. The ECMO turnaround time was very short during tracheal resection and anastomosis. Protamine injection was used to neutralize heparin immediately after the removal of ECMO, so there would be no large blood loss during the operation. 2) There are many intubation modes that can be selected during ECMO operation, such as femoral arteriovenous intubation, internal jugular vein intubation, and common carotid artery intubation, which provide multiple anesthesia modes. 3) V-V ECMO is used to maintain a satisfactory oxygen supply without endotracheal intubation. 4) ECMO reduces the impact of tracheal intubation on the surgical field in a narrow space, improves the accuracy of anastomosis, and clearly avoids injury to the recurrent laryngeal nerve during anastomosis. There are also disadvantages in this case: ECMO does increase the cost of treatment, and the operation time will increase accordingly. However, we believe that under the condition of ECMO, the visual field of end-to-end tracheal anastomosis is more sufficient, and the anastomosis time can be greatly shortened, which actually shortens the operation time in another aspect.

In conclusion, this case proves that using ECMO for thyroid carcinoma resection, invaded trachea resection, and trachea reconstruction is safe and effective, which reduces the obstruction of endotracheal intubation on the operative field, guarantees the rapid and efficient end-to-end anastomosis in the upper trachea, and clearly avoids laryngeal recurrent nerve injury in the process of anastomosis, which provides another reference anesthesia and surgical method.

## Data availability statement

The raw data supporting the conclusions of this article will be made available by the authors, without undue reservation.

## Ethics statement

Written informed consent was obtained from the individual(s) for the publication of any potentially identifiable images or data included in this article.

## Author contributions

BH, SZ, LR, and YZho proceed the surgery. These authors contributed equally to this work and share first authorship. QC, JT, and YZha: assistant in nursing. MT and YQ prepared the first draft of the manuscript. HW finalized the manuscript and instructed the case. All authors contributed to the article and approved the submitted version.
